# Investigating Effects of IR-780 in Animal Models of B16-F10 Melanoma: New Approach in Lung Metastasis

**DOI:** 10.3390/molecules28196942

**Published:** 2023-10-05

**Authors:** Evelyn de Andrade Salomão, Valter Aragão do Nascimento, Caio Fernando Ramalho de Oliveira, Iandara Schettert Silva, Rita de Cássia Avellaneda Guimarães, Danielle Bogo

**Affiliations:** 1 Graduate Program in Health and Development in Central-West Region, Federal University of Mato Grosso do Sul (UFMS), Campo Grande 79070-900, Brazil; aragao60@hotmail.com (V.A.d.N.); rita.guimaraes@ufms.br (R.d.C.A.G.); danielle.bogo@ufms.br (D.B.); 2 Federal Institute of Mato Grosso, Guarantã do Norte 78520-000, Brazil; caio.oliveira@ifmt.edu.br; 3 Federal University of Mato Grosso do Sul (UFMS), Campo Grande 79070-900, Brazil; ian.da.ra@hotmail.com

**Keywords:** biomarker, fluorescence, in vivo imaging

## Abstract

IR-780 is a fluorescent marker, photostable and non-toxic, and is widely used in tumor targeting; however, studies on the impact of IR-780 in animal models of B16-F10 melanoma are scarce in the literature. Therefore, this study aims to analyze behavior of this marker in melanoma cells using in vitro and in vivo analyses with fluorescence microscopy to conduct an analysis of cell culture, and an in vivo imaging system for an analysis of cell culture, tumor targeting on animals, and organ examination. In vitro analysis showed that B16-F10 cells at a concentration of 2 × 10^5^ cells.plate^−1^ allowed a better visualization using 20 μM of IR-780. Furthermore, the location of IR-780 accumulation was confirmed by its fluorescence microscopy. Through in vivo studies, fluorescence was not observed in subcutaneous nodules, and it was found that animals that received intraperitoneal injection of B16-F10 cells presented ascites and did not absorb IR-780. Additionally, animals exhibiting lung metastasis showed fluorescence in ex vivo lung images. Therefore, use of the IR-780 marker for evaluating the progression of tumor growth did not demonstrate efficiency; however, it was effective in diagnosing pulmonary metastatic tumors. Although this marker presented limitations, results of evaluating pulmonary involvement through ex vivo fluorescence imaging were determined based on intensity of fluorescence.

## 1. Introduction

Melanoma skin cancer is considered a serious disease due to its high possibility of metastasis. If detected in its early stages, melanoma has a favorable prognosis. However, when metastasis is diagnosed, the patient’s five-year survival rate is 31.9% [1,2]. To understand the development or treatment of various types of cancer in humans, in recent years animal models are the most used to produce reliable data with clinical translation [3]. In this case, in both human and animal models, detection of tumor biomarkers in body fluids is the most non-invasive way to identify the developmental stage of a tumor [4]. In this way, fluorescence markers are widely used in medicine for cancer diagnoses [5,6,7]. A good marker should have certain characteristics such as low toxicity, short half-life, and fluorescence in a sufficient period for reliable diagnosis [8].

In vitro studies with the B16-F10 melanoma cell lineage generally use markers such as DAPI, CD31 (immunofluorescent monoclonal antibody) [9], Methylene blue [10], Phalloidin-Fluor 488, and Deep Red Plasma Membrane Stain [11], among others. They also perform cellular modifications by adding plasmids containing the GFP (green fluorescence protein) gene [12] or luciferase [13]. However, there are few studies that present in vivo or ex vivo tumor monitoring of B16-F10 using an in vivo imaging system, especially using the IR-780 marker.

This marker is a lipophilic cation with a molecular weight of 667 Da, which is photostable and non-toxic [14,15,16]. Its selective mitochondrial uptake is energy-dependent, membrane potential-dependent, and facilitated by organic anion-transporting peptides (OATPs) [17]. Plasma proteins also play an important role in the marker’s uptake by tumor cells. IR-780 has the ability to form non-covalent complexes with plasma albumin, which enhances its fluorescence capacity [18].

IR-780 has been used in a wide variety of tumor cells and in vivo studies, such as in cancer cell lineages of human renal adenocarcinoma (786-O; ACHN) in nude mice [19]; human breast adenocarcinoma (MCF-7), human cervix adenocarcinoma (HeLa) and human osteosarcoma (MG-63) cells in athymic nude mice [20]; mouse mammary carcinoma (4T1) in BALB/c mice [21,22] and nude mice [23]; human glioblastoma (U-87 MG) in athymic nude mice [24]; human colon carcinoma (HCT 116) and adenocarcinoma (SW 620) in nude mice [25]; human melanoma (SK-Mel-28) in nude BALB/c mice [26]; mouse lung carcinoma (LL/2) in BALB/c athymic nude mice [27]; human pancreatic carcinoma (MIA-Pa-Ca2) in BALB/c nude mice [28]; and human lung adenocarcinoma (NCI-H3122) and MCF-7 in immune-deficient nude mice [29]. In addition to being used as a fluorescence marker for in vivo tumor detection, IR-780 is also used in photothermal therapies due to its ability to generate heat and reactive oxygen species (ROS) [30].

The aim of the study was using a new approach to evaluate the development of a murine melanoma tumor of B16-F10 cell line in BALB/c mice and investigate the effects of using the tumor marker IR-780 in vitro and in vivo tests in three animal models using the In-Vivo Xtreme imaging system.

## 2. Results

### 2.1. In Vitro IR-780 Uptake in B16-F10

**Figure 1:**
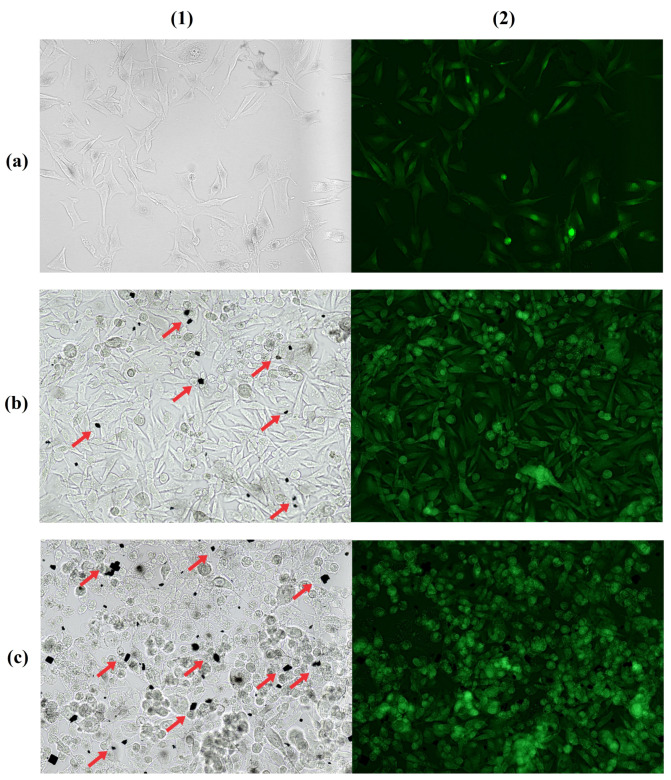
Bright field (**1**) and fluorescence microscopy (**2**) of cell concentration 2 × 10^5^ cells with 20 µM IR-780 (**a**); 4 × 10^5^ cells with 40 µM IR-780 (**b**); and 4 × 10^5^ cells with 80 µM IR-780 (**c**). Red arrows indicate excess dye crystals at highest concentrations of IR-780. Obtained with Leica DM 2000 Led fluorescence microscope (200×).

From cellular analysis, the concentration of 2 × 10^5^ allowed for better visualization by presenting a defined monolayer, without cell overlap (Figure 1(a1)). The concentrations of 40 μM and 80 μM of IR-780 presented small dye crystals, meaning that their solubility was compromised, resulting in saturation of medium (Figure 1(b1,c1)). At the highest concentration (80 μM), it was observed that cells entered in apoptosis in approximately 10 min, resulting in a gradual loss of fluorescence. Therefore, a cell concentration of 2 × 10^5^ with 20 μM of IR-780 was considered more effective for the analyses.

**Figure 2:**
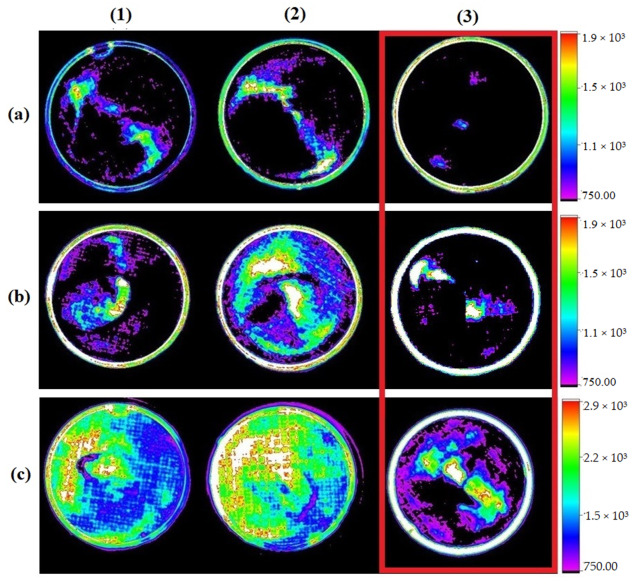
Image showing fluorescence emitted by the addition of IR-780 at concentrations of 20 µM (**1**), 40 µM (**2**), and 80 µM (**3**) in different cell concentrations: 1 × 10^5^ (**a**), 2 × 10^5^ (**b**), and 4 × 10^5^ (**c**). Red highlight on highest concentration of IR-780 showing smaller areas of fluorescence. Obtained with In-Vivo Xtreme, Bruker.

Images obtained through In-Vivo Xtreme demonstrated results similar to those observed in fluorescence microscopy. In this case, for the concentration of 80 μM of IR-780 (Figure 2(a3,b3,c3)) it was observed that the image presented fluorescence, but in smaller areas when compared to concentrations of 20 μM and 40 μM (Figure 2(a1,a2,b1,b2,c1,c2)), corroborating the information that apoptosis interferes with the emission of fluorescence.

### 2.2. In Vitro IR-780 Accumulation in B16-F10

**Figure 3:**
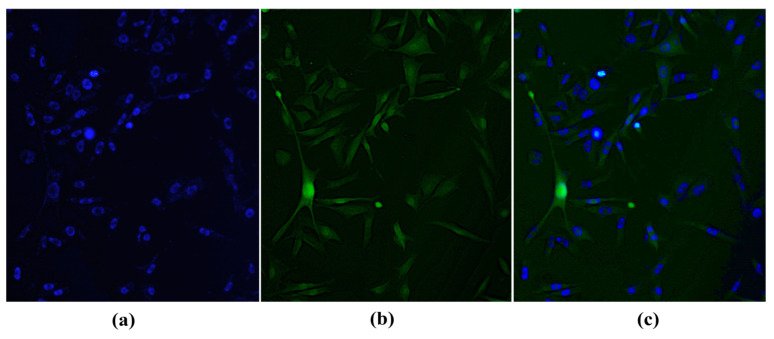
B16-F10 cell with its nuclei stained with Hoechst 33342 (**a**); cytosol stained with IR-780 (**b**); and overlaid images showing the difference between nucleus and cytosol (**c**). Obtained with Leica DM 2000 Led fluorescence microscope (200×).

Hoechst 33342 nuclear dye was used as a contrast effect. In this case, the location of IR-780 accumulation was confirmed through its delimited fluorescence in the cellular cytosol (Figure 3b).

### 2.3. In Vivo Models

**Figure 4:**
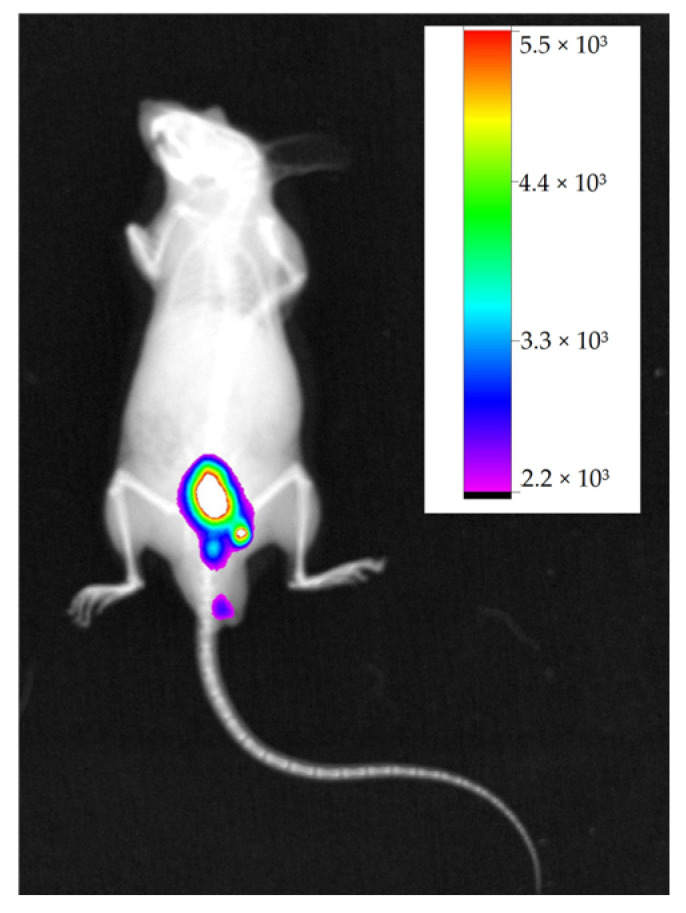
Negative control animal showing fluorescence in the caudal abdominal region and its fluorescence intensity scale. Obtained with In-Vivo Xtreme, Bruker.

For comparative purposes of tumor experimental models, a control animal was used under the same study conditions as the experimental groups, without receiving tumor cells, only with IR-780 marker. However, the tumor-free animal presented fluorescence in caudal abdominal region (Figure 4).

#### 2.3.1. Subcutaneous Melanoma

**Figure 5:**
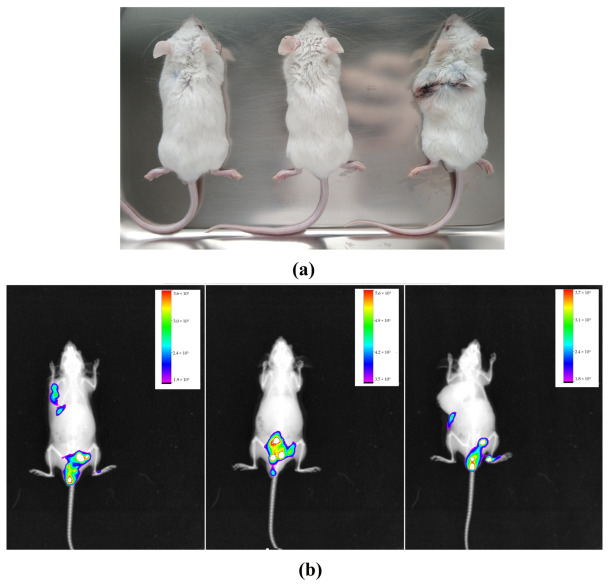
Animals showing visible dorsal subcutaneous nodules (**a**); images of animals in In-Vivo Xtreme image system with their respective fluorescence intensity scales (**b**). The images show higher fluorescence intensity in the caudal abdominal region and little or no fluorescence in the subcutaneous nodule region.

The animals subjected to subcutaneous injection of B16-F10 cells in the dorsal region developed nodules within 25 days (Figure 5a). After intraperitoneal administration of IR-780 in animals, fluorescence images were acquired using the In-Vivo Xtreme image system, which it was found through obtained fluorescence results that the caudal abdominal region presented the highest peak of fluorescence; on the other hand, the region near the nodules showed little or no fluorescence when evaluating the overall image of animal (Figure 5b).

**Figure 6:**
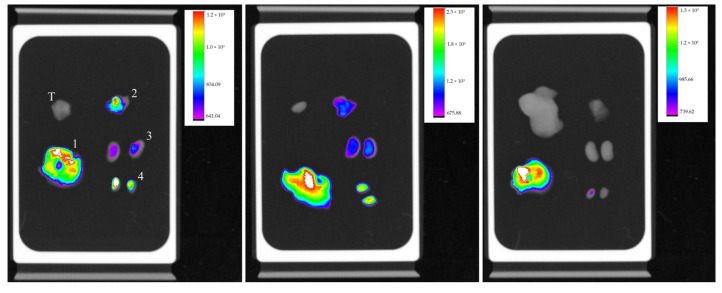
Organ’s fluorescence image of three animals with their respective scales of fluorescence intensity acquired by the equipment In-Vivo Xtreme image system; liver (1), lungs (2), kidneys (3) testicles (4), and tumor (T), at the same sequence.

The organs of these animals were also analyzed by fluorescence, along with already-sectioned tumors. Livers and kidneys were analyzed because they are organs of metabolism and excretion, testicles because they are close to the areas of highest fluorescence intensity, and lungs because they are considered a target organ for metastasis when it comes to B16-F10 lineage. As expected, livers presented higher fluorescence intensity, but no tumor was able to emit significant fluorescence (Figure 6).

#### 2.3.2. Pulmonary Metastasis: Intraperitoneal Inoculation

**Figure 7:**
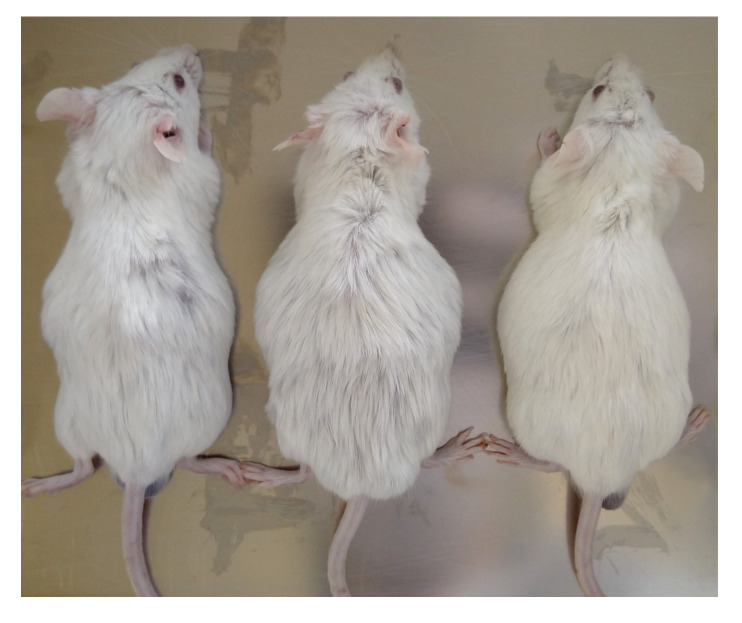
All specimens in the group presented abdominal distension, compatible with ascites.

The animal model used for development of lung metastasis with intraperitoneal injection of tumor cells was not favorable for the study. On the thirtieth day, all three specimens showed abdominal swelling, as shown in Figure 7, compatible with ascites. However, this period was not sufficient for metastasis development.

**Figure 8:**
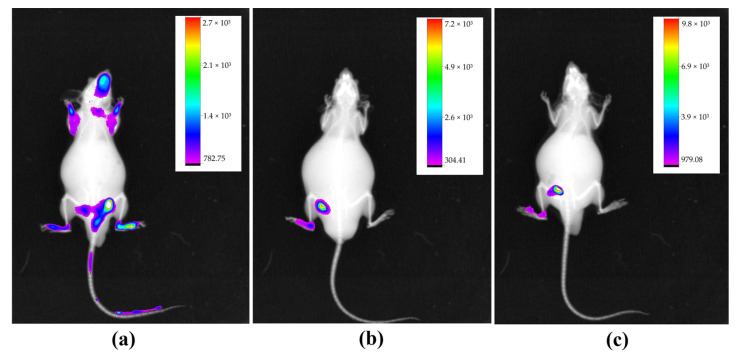
All animals in the group presented abdominal distension, compatible with ascites. One animal presented multiple areas of fluorescence (**a**), while animals with more pronounced ascites presented limited fluorescence images (**b**,**c**). Obtained with In-Vivo Xtreme, Bruker.

**Figure 9:**
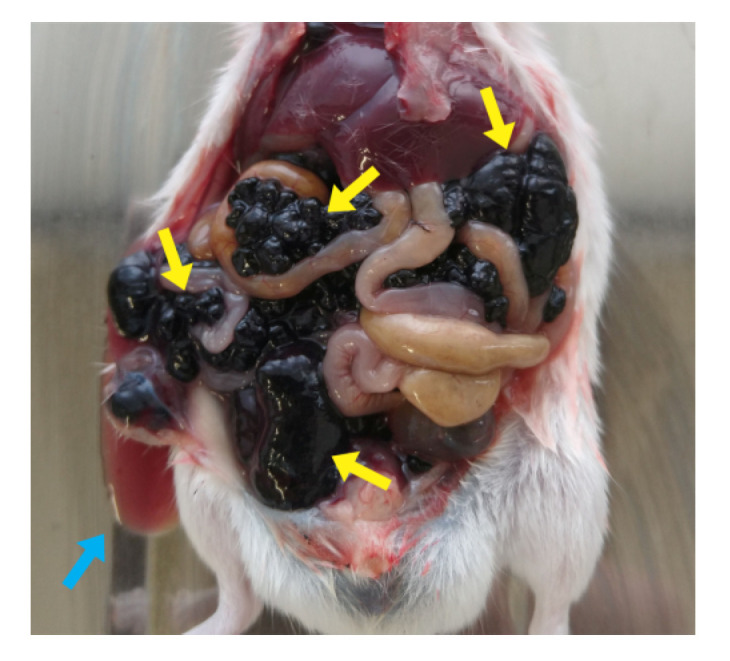
Abdominal necropsy. Several tumor nodules disseminated throughout mesentery are evidenced by yellow arrows; blue arrow demonstrates the ascitic fluid.

Images obtained from the In-Vivo Xtreme image system show a small area with higher fluorescence intensity in caudal abdominal region. Only in one animal were several areas of lower fluorescence intensity observed (Figure 8a), while animals with more pronounced ascites did not present disseminated fluorescence (Figure 8b,c). During necropsy, it was observed that none of animals had macroscopically visible lung metastases, but all had numerous tumor nodules widely disseminated throughout the abdominal mesentery (Figure 9).

**Figure 10:**
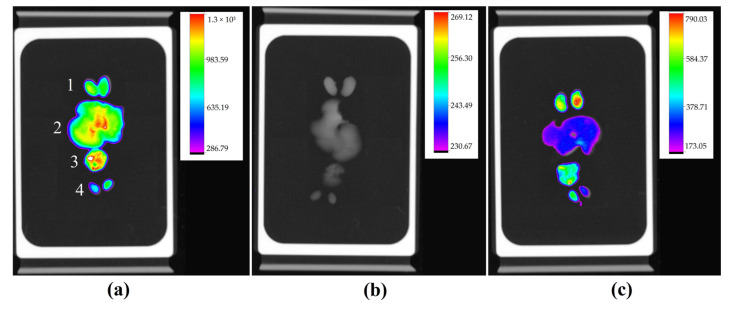
Organ fluorescence image of three animals with their respective scales of fluorescence intensity acquired by the In-Vivo Xtreme image system: kidneys (1), liver (2), lungs (3), and testicles (4), at the same sequence. Animal with a lower amount of ascitic fluid presenting higher fluorescence (**a**), animal with higher presence of ascites without fluorescence labeling (**b**), and animal with moderate ascites presenting low fluorescence intensity (**c**).

For the same reason as described earlier in the subcutaneous model, the chosen organs for analysis were liver, kidneys, lungs, and testicles. Animals with the highest amount of ascitic fluid did not show fluorescence in organs, indicating the possible difficulty in absorbing IR-780 (Figure 10b). The other two animals showed fluorescence in all organs, but the animal with less ascites showed a higher intensity of fluorescence (Figure 10a).

#### 2.3.3. Pulmonary Metastasis: Intravenous Inoculation

**Figure 11:**
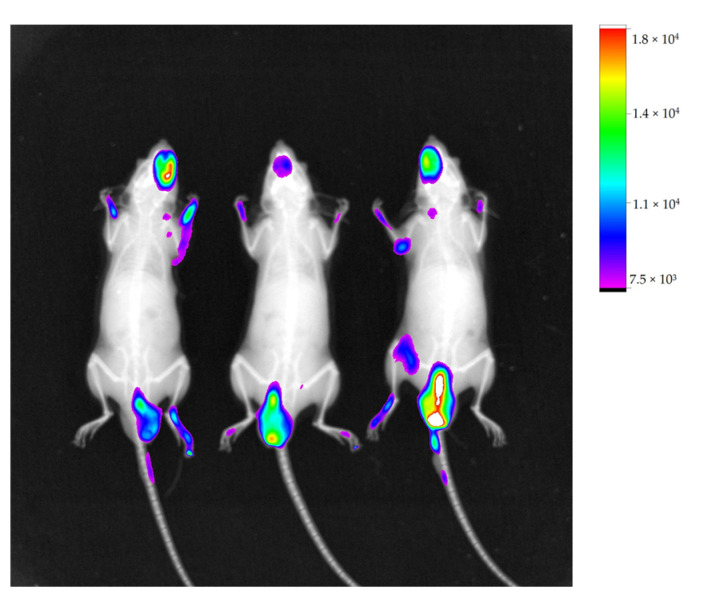
Image of animals with their respective fluorescence intensity scale, acquired with In-Vivo Xtreme image system. Showing regions of fluorescence in caudal abdominal region and some points in cranial region.

The animals used as a model of lung metastasis by intravenous inoculation of B16-F10 cells were followed for 30 days, when they began to show behavioral changes such as lethargy and reduced food intake. As in other groups, IR-780 was administered prior to imaging, which showed the same pattern of fluorescence localized in the caudal abdominal region, with some points of fluorescence in the cranial region and no points in the lungs, a region of interest (Figure 11).

**Figure 12:**
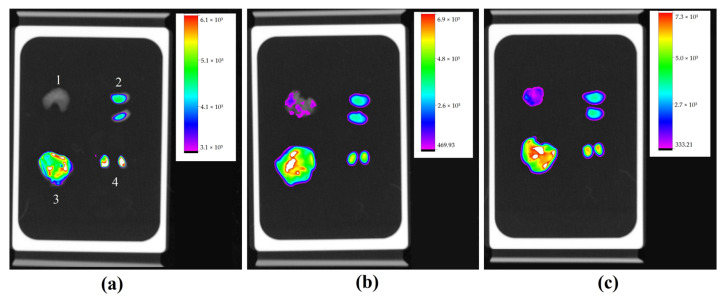
Fluorescence image of organs of three animals with their respective scales of fluorescence intensity acquired by equipment In-Vivo Xtreme image system; lungs (1), kidneys (2), liver (3), and testicles (4), at the same sequence. Lung with more nodules showing no fluorescence (**a**), lung with significant nodules with mild fluorescence (**b**), and less affected lung with higher fluorescence (**c**).

Maintaining the same evaluation criteria as the other groups, organs analyzed for the second model of lung metastasis were also the lungs, kidneys, testicles and liver. Images obtained by In-Vivo Xtreme showed that the lung with the most compromised surface by nodules did not show fluorescence (Figure 12a). The fluorescence image of other lungs was inversely proportional to number of superficial nodules, meaning that least compromised lung showed highest fluorescence (Figure 12c).

### 2.4. Metastatic Lung Macroscopic Evaluation

**Figure 13:**
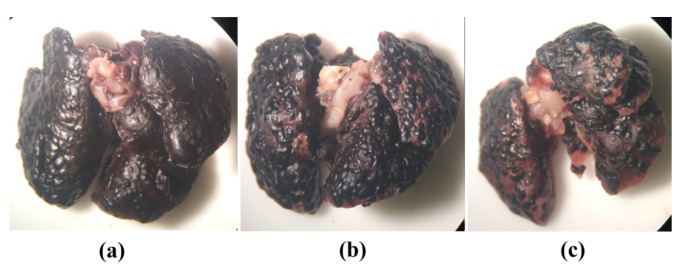
Macroscopic image of lung most affected by numerous malignant tumor nodules on pleural surface (**a**); lung with high involvement (**b**); lung with lower involvement (**c**).

In a group with metastasis induced by intravenous injection of tumor cells, the model of pulmonary metastasis was confirmed by macroscopic evaluation of lungs, where the presence of tumor nodules on the entire pleural surface of lungs was evident, as well as the difference in involvement between each lung. It was clear that Figure 13a was more involved compared to Figure 13b, which in turn was more involved than Figure 13c, corroborating with results obtained in fluorescence analysis.

### 2.5. Microscopic Evaluation

**Figure 14:**
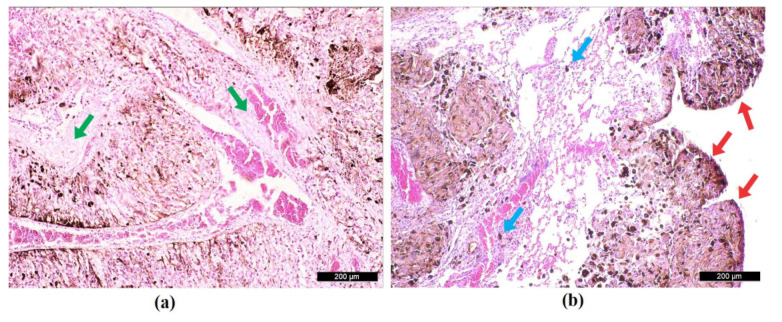
Histological image of the lung parenchyma with green arrows highlighting a few areas of intact tissue surrounded by metastatic tumor nodules (**a**), histological image of the lung pleura with red arrows showing nodules throughout the pleural surface and blue arrows demonstrating isolated foci of metastasis in lung parenchyma (**b**).

Metastatic lungs were used for histological analysis, which did not indicate any structural, inflammatory, or necrotic compromise. However, the tumor implantation percentage was high, with involvement of more than 90% of parenchyma lung (Figure 14a) and 99% of pleural surface (Figure 14b).

Histological analyses were also performed on organs that showed higher fluorescence to determine if there was any cellular implantation. However, intact tissue without signs of abnormal cell growth or inflammatory and necrotic processes was observed. Thus, it can be stated that there was no metastasis to other organs (images not presented).

## 3. Discussion

Our research demonstrates that a dose of 80 μM of IR-780 induces cellular apoptosis in approximately 10 min, corroborating with findings presented by Lu et al. [31] that IR-780 has apoptotic activity in glioblastoma cells observed through flow cytometry. Other studies using IR-780 at 15 μM in human bladder adenocarcinoma cells [32] and 10 μM in two lines of renal adenocarcinoma cells [19] also present an apoptotic effect. Therefore, with microscopic and radiographic analyses presented in our study, the concentration of 20 μM of IR-780 was sufficient to obtain labeling without causing cellular damage, and the concentration of 2 × 10^5^ cells showed better confluence for microscopic cellular analysis. The accumulation of IR-780 in cellular cytosol corroborates with information from other studies that have demonstrated its affinity for mitochondria [17,33,34].

Studies also have shown the interaction of the IR-780 marker with other molecules in order to form stable nano-complexes or micelles with ability to selectively amplify cytotoxic activity in tumor cells. Some of these interactions can occur with organic compounds, such as albumin, with which it forms weak non-covalent binding [18,23,35,36]. This kind of bind explains fluorescence images in animals with no tumor cells (Figure 4), where non-fluorescence images were expected.

In in vivo analysis, the present study used 0.45 mg·kg^−1^ of IR-780 intraperitoneally in BALB/c mouse strain without presenting any toxicological alterations to the animal. Zhang et al. [16] used 0.2 mg·kg^−1^ intravenously in athymic nude mice and Shen et al. [32] administered 3 mg·kg^−1^ intraperitoneally in mice; in both studies no toxicological alterations were reported. However, Jiang et al. [36] demonstrated IR-780 toxicity by administering 2.5 mg·kg^−1^ intravenously in BALB/c mice, resulting in 100% lethality. Therefore, the route of administration is a crucial factor in determining toxicity, as the concentration near 2.5 mg·kg^−1^ administered intravenously was lethal, while higher concentration of 3 mg·kg^−1^ administered intraperitoneally did not present any alterations in animals.

Use of fluorescence marker IR-780 in B16-F10 cells is rarely reported in literature. According to results obtained in our study, use of this marker for evaluation of subcutaneous tumor growth progression did not demonstrate efficiency, as obtained images were not focused on the tumor, as also shown by He et al. [37] who did not observe fluorescence in subcutaneous melanoma nodules using the same marker.

As observed, melanoma tumors have a dark coloration due to presence of melanin in large quantities and for this reason, the capture of fluorescence intensity by equipment is compromised, as melanin absorbs a broad spectrum of light [38]. For this reason, fluorescence images of subcutaneous nodules did not present results.

Low absorption of IR-780 in animals presenting ascites can be explained due to increased intraperitoneal pressure. This abdominal distension caused by inflammatory fluid has systemic repercussions causing several alterations, but mainly reduces blood flow and blocks lymphatic system of region; therefore, the marker absorption is reduced or does not occur [39,40].

Results of evaluation of lung involvement through ex vivo fluorescence images were defined and corroborated by histological and macroscopic analyses. As mentioned previously, the presence of melanin competes for absorption of light; therefore, this result should be interpreted as the higher fluorescence the intensity, the lower the tissue involvement. On the other hand, the lower the fluorescence intensity, the greater the tissue involvement by the tumor [41].

## 4. Materials and Methods

### 4.1. Cell Lines and Culture

Murine melanoma (B16-F10) was maintained in DMEM (Dulbecco’s Modified Eagle Medium) Sigma-Aldrich (São Paulo, Brazil) with 10% of fetal bovine serum (GibcoTM) Thermo Fisher (São Paulo, Brazil) and 1% streptomycin/penicillin (100 μg·mL^−1^ and 100 UI·mL^−1^, respectively) Sigma-Aldrich, kept at 37 °C and atmosphere with 5% CO_2_.

### 4.2. IR-780 Iodide, Sigma-Aldrich

To stock solution of IR-780 iodide, 0.01 g·mL^−1^, diluted in DMSO Sigma-Aldrich, stored at 4 °C away from direct light.

### 4.3. Fluorescence Analysis

The equipment used for fluorescence analyses of cell culture was a Leica DM 2000 Led fluorescence microscope with LAS V4.12 software. For analysis of cell culture and tumor targeting on animals and organs an In-Vivo Xtreme BI 4MP Bruker (Billerica, MA, USA) was used, with the foreground in fluorescence mode, a multi-wavelength light source, with the excitation filter at 760 and emission at 830, camera controls with standard exposure type, high speed mode, exposure time 2.0 s, Bin 2 × 2 pixels, FOV 19 cm, fStop 1.1, focal plane 0 mm and histogram in rainbow model. The background was in X-ray mode, with kVp 45, exposure time of 0.1 s, Bin 1 × 1 pixel, fStop 2.8, focal plane 0 mm and grayscale histogram, using a Bruker Molecular Imaging software.

### 4.4. In Vitro IR-780 Uptake in B16-F10

The B16-F10 cell line was grown in Petri dishes for cell culture (32.8 mm with grid) at concentrations of 1 × 10^5^, 2 × 10^5^ and 4 × 10^5^ cells·plate^−1^, incubated for 24 h. Each cell concentration received IR-780 at concentrations of 20 μM, 40 μM, and 80 μM diluted in DMEM without fetal serum, from the stock solution, totaling nine plates. The plates were incubated for a further 4 h and after were carefully aspirated and washed with PBS. This process was three times repeated. Finally, cells were observed in a fluorescence microscope and in the In-Vivo Xtreme imaging system.

### 4.5. In Vitro IR-780 Accumulation in B16-F10

To determine the location of dye accumulation, a Petri dish (32.8 mm) with 2 × 10^5^ cell·plate^−1^, 20 μM of IR-780, and a drop of Hoechst 33342 (NucBlueTM) Thermo Fisher, was used. Cells were observed in a fluorescence microscope.

### 4.6. Experimental Animals

Mus musculus BALB/c, males, 25–35 g, 4–6 weeks old, were purchased from the Central Animal Facility linked to Dean of Research and Graduate Studies of UFMS. Animals were kept in mini isolators of the Basic Alesco^®^ Ventilated Rack (São Paulo, Brazil), at temperature and relative humidity of 21 °C and 60%, 12 h light/dark cycle, receiving standard chow (NUVILAB^®^ CR) and water ad libitum.

#### In Vivo Models

To perform three tumor models, B16-F10 cells (5 × 10^5^) were diluted in different final volumes according to the used technique: 200 μL for subcutaneous (SC) and intraperitoneal (IP) injections, and 50 μL to intravenous (IV) injection. Each group was composed of 3 mice (n = 3).

To approach results, one animal was used as a negative control for IR-780. This animal was maintained under the same conditions but did not receive injection of tumor cells. On the last day of the experiment, this animal was intraperitoneal inoculated with 200 μL IR-780 0.45 mg·kg^−1^, prepared from stock solution in 0.9% saline. Images were obtained within 24 h, using inhalational anesthetic isoflurane for animal containment.

Ex vivo images of the subcutaneous nodules and the organs lung, kidney, liver, and testicle were also obtained.

a. Subcutaneous melanoma: To obtain subcutaneous nodule, cells were administered SC in a single dose at dorsal region of neck. After 24 days of cell application, animals received 200 μL IR-780 as control animal and the other groups.

b. Pulmonary metastasis (intraperitoneal): The first metastatic model was performed by single administration of cells by IP in lower abdominal quadrant. After 29 days, animals received IR-780.

c. Pulmonary metastasis (intravenous): For the second metastatic model, cells were administered by IV into lateral tail vein, using isoflurane to contain animal. These animals also received IR-780 after 29 days.

### 4.7. Metastatic Lung Macroscopic Evaluation

Metastatic nodules on the pleural surface of the lungs were evaluated using an Olympus optical stereomicroscope.

### 4.8. Microscopic Evaluation

Two sections from complete left lung lobe, liver, kidney, and testicles stained with HE was used for quantitative analysis using the Leica DM 2000 Led microscope and LAS V4.12 software. Total area of irregular cell growth and total area of analyzed organs were used to calculated implantation percentage of metastasis.

## 5. Conclusions

In this study, we tested in vitro and in vivo the effect of IR-780 marker on B16-F10 melanoma cells. The microscopic and radiographic analyses presented in our study using IR-780 revealed that a concentration of 20 μM did not cause any cellular damage, and a cell concentration of 2 × 10^5^ showed better confluence for microscopic analysis. Fluorescence images of an animal without tumor cells were observed, possibly due to binding of marker with other organic molecules. Additionally, use of IR-780 for evaluating subcutaneous tumor growth evolution did not demonstrate efficiency. However, for pulmonary metastatic tumors, it was effective in diagnosis. Although IR-780 presented limitations in this study, results of evaluating pulmonary involvement through ex vivo fluorescence images were defined and corroborated by histological and macroscopic analyses, and should be interpreted considering fluorescence intensity.

Although some in vivo analyses showed limitations, further studies should be carried out involving this marker with melanoma cells. Since IR-780 has apoptotic properties and is described in the literature as an adjuvant in cancer chemo-phototherapy due to its optical properties and capacity to generate ROS, its use as an active drug against melanoma cells should be deepened.

## Data Availability

The data used to support the findings of this study are available from the corresponding author upon request.
